# Now, more than ever, it’s time to address the neglect of female genital schistosomiasis

**DOI:** 10.1017/S0031182025101297

**Published:** 2025-12

**Authors:** Fiona M. Fleming, Ashley Preston, Anthony Kerkula Bettee, Norbert Dje, Victoria Gamba, Anouk Gouvras, Margaret Gyapong, Julie Jacobson, Christine Kalume, Karsor K. K. Kollie, Alain-Claver Kouamin, Alison Krentel, Elizabeth F. Long, Humphrey Deogratias Mazigo, Makia Christine Masong, Akinola Stephen Oluwole, Leora Pillay, Ibrahim Rabiu, Bodo S. Randrianasolo, Florence Wakesho, Yael Velleman

**Affiliations:** 1 Unlimit Health, London, UK; 2 Ministry of Health and Social Welfare, Monrovia, Liberia; 3 Programme National de Lutte Contre les Maladies Tropicales Négligées à Chimiothérapie Préventive, Ministre de la Santé, de l’Hygiène Publique et de la Couverture Maladie Universelle, Abidjan, Côte d’Ivoire; 4 Kenya Obstetrical and Gynaecological Society, Nairobi, Kenya; 5 Global Schistosomiasis Alliance, London, UK; 6 Institute of Health Research, University of Health and Allied Sciences, Ho, Ghana; 7 Department of Global Health, Georgetown University, Washington DC, DC, USA; 8 Bridges to Development, Seattle, WA, USA; 9 Frontline AIDS, Brighton, UK; 10 School of Epidemiology and Public Health, University of Ottawa, Ottawa, Canada; 11 Buyère Health Research Institute, Ottawa, Canada; 12 Coalition for Operational Research on Neglected Tropical Diseases, Decatur, GA, USA; 13 Catholic University of Health and Allied Sciences, Mwanza, Tanzania; 14 Catholic University of Central Africa, Yaoundé, Cameroon; 15 Sightsavers, Abuja, Nigeria; 16 Gombe State University, Gombe, Nigeria; 17 Association K’OLO VANONA, Antananarivo, Madagascar; 18 Ministry of Health, Nairobi, Kenya; 19 University of Nairobi, Nairobi, Kenya

**Keywords:** cervical cancer, female genital schistosomiasis, global health policy, health service integration, HIV, HPV, neglected tropical diseases, sexual and reproductive health, sustainable resourcing, women’s health

## Abstract

Female genital schistosomiasis (FGS) remains a neglected sexual and reproductive health (SRH) condition, predominantly affecting women and girls in sub‐Saharan Africa. Infection with *Schistosoma haematobium*, resulting in trapped parasite eggs in the genital tract, causes lesions that mimic sexually transmitted infections and cervical neoplasia, often leading to misdiagnosis, stigma and delayed treatment. This review summarises current developments on FGS burden, prevention, diagnostics, integration, policy, community engagement and identifies critical threats to progress. Ongoing surveys show promise in ensuring robust burden estimates and age‐related risk data. Diagnostic advances include portable colposcopy, digital image analysis techniques and molecular assays, although limitations persist in resource‐limited settings. Praziquantel remains the cornerstone of treatment, yet single‐dose regimens inadequately reverse established lesions; repeated dosing shows improved parasite clearance but limited lesion regression, highlighting the necessity for early, life‐course preventive chemotherapy including access to paediatric praziquantel. Successful programmatic pilots have developed training curricula, minimum service packages, community engagement tools and have integrated FGS care into SRH platforms. Policy momentum is building through World Health Organization taskforces and national strategies, yet sustainable financing remains a challenge. Key threats include bilateral aid reductions, climate change, emerging infections, rising healthcare costs and persistent gender inequities. To address these challenges, we propose seven priority actions, encompassing all health system building blocks, for the global community. Nationally coordinated, multisectoral efforts are urgently required to embed FGS prevention, diagnosis and management within broader health systems, thereby improving outcomes for affected women and girls.

## Graphical abstract

**Figure:**
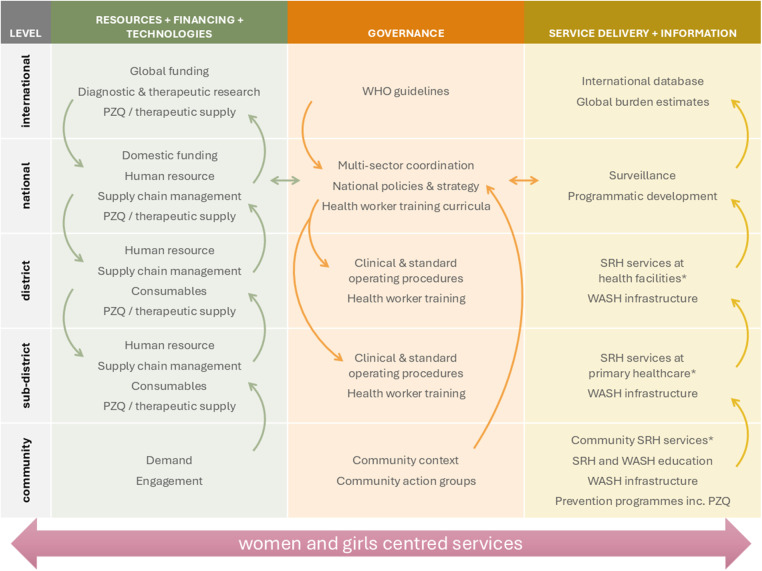

## Introduction

Female Genital Schistosomiasis (FGS) is a neglected gynaecological condition with severe sexual and reproductive health (SRH) complications (Jacobson et al. 2022). It is also considered a cofactor in the acquisition of human immunodeficiency virus (HIV) and human papillomavirus (HPV) infections (Engels et al. 2020; Sturt et al. 2020a). Up to an estimated 56 million women and girls, primarily in sub-Saharan Africa, are at risk of this condition, most of whom lack access to safe water, healthcare services, and targeted prevention programmes (Hotez et al. 2019). Parasitic *Schistosoma haematobium* worms cause the urogenital form of the waterborne disease known as schistosomiasis. FGS results from a chronic inflammatory reaction of the host to the highly antigenic *S. haematobium* eggs that become trapped in the tissues of the female genital tract (Colley et al. 2014; Rossi et al. 2024). If left untreated, the resulting disease may progress, causing irreversible genital lesions and impacting fertility, for which no validated therapeutic options currently exist (Arenholt et al. 2024). Clinically, FGS may be mistaken for cervical cancer and often mimics the symptoms of sexually transmitted infections (STIs), such as pelvic pain, abnormal vaginal discharge, post-coital bleeding, and genital itching or burning (Kaizilege et al. 2022).

Consequently, FGS is frequently misdiagnosed as an STI. As a result, the affected women and girls may face stigma that leads to reduced care-seeking behaviour, intra-familial conflict and gender-based violence, which have significant impacts on their mental health and well-being (Schuster et al. 2022). These challenges are compounded by the lack of FGS integration into SRH and broader services for women’s health rights, alongside limited access to effective diagnostic tools, often resulting in delayed or inappropriate treatment and poor health outcomes (Lamberti et al. 2024).

Women and girls in endemic communities are exposed to *S. haematobium* infection through routine water contact during activities such as bathing, washing clothes, fetching water, farming, or fishing (Colley et al. 2014; Buonfrate et al. 2025). Schistosomiasis prevalence is highest in communities with inadequate access to safe water and sanitation, where reliance on infested natural water sources is common. Thus, improved access to a safe, reliable water supply and sanitation infrastructure is critical to reducing transmission. Mass drug administration (MDA) of praziquantel is a key public health intervention aimed at treating schistosomiasis and preventing FGS and other morbidities by eliminating the parasites before genital complications develop (UNAIDS, 2019; World Health Organization, 2022). However, current MDA strategies predominantly target school-aged children and have not been sufficient to address FGS as a broader public health concern. Therefore, additional targeted interventions across the life-course are urgently needed (Preston et al. 2023).

## Global response to FGS

Since the first World Health Organization (WHO) consultation on FGS in 2009 and an international workshop held in Johannesburg in 2015, progress for integrating FGS management into SRH services has remained limited, despite the compelling rationale for doing so (World Health Organization, 2010; BRIGHT Academy, 2015; Hotez et al. 2019). High-level advocacy – including the seminal WHO and UNAIDS joint publication calling for FGS and HIV integration – and renewed interest and funding following the global FGS meeting in Liverpool have yet to translate into large-scale interventions (Global Schistosomiasis Alliance, 2019; UNAIDS, 2019; Engels et al. 2020). To date, most efforts have been confined to small-scale research projects and pilot initiatives.

There has been a growing consensus on the need for a more coordinated response (Hotez et al. 2019; Sturt et al. 2020a; Engels et al. 2020; Jacobson et al. 2022; World Health Organization, 2022). This includes the consolidation of existing research findings, standardisation of diagnostic tools, and the formulation of practical, actionable recommendations that health ministries can adopt to inform national strategies (Krentel et al. 2024; Lamberti et al. 2024; Pillay et al. 2024; Mberu et al. 2025). A critical challenge remains that FGS prevalence is primarily inferred from *S. haematobium* infection data, rather than from FGS-specific surveillance. Nevertheless, surveillance is hampered by the major gaps in routine data collection, diagnostic availability, integration of FGS into routine national health management information systems (HMIS), therapeutic options and community engagement activities, all of which urgently require attention.

In the current context of constrained global health financing, competing priorities and threats to women’s and girls’ rights and programmes including emerging infectious diseases, now more than ever, is the time to leverage synergies with established SRH and broader health systems and maximize cross-sectoral resource-sharing. This review offers a timely opportunity to assess the current momentum and identify the strategic actions necessary to close remaining gaps.

### Determining the burden of FGS

Significant progress is being made to address evidence gaps in the burden of FGS through the ongoing Multi-Country Assessment of Prevalence for FGS (MAP-FGS) study (COR-NTD, 2025). This research aims to generate critical data on FGS prevalence and to investigate age-related trends by exploring risk factors such as *S. haematobium* infection, previous treatment exposure and coinfections with HPV and STIs (gonorrhoea and trichomoniasis). The study includes women and girls aged 10 to 60 years, with a secondary aim to develop practical, age-appropriate diagnostic approaches for use in endemic settings. Data from six countries – Ghana, Mali, Tanzania, Madagascar, Senegal and Nigeria – will be analysed to estimate FGS prevalence across archetypal transmission settings. These findings will be used to model the first robust regional estimates of FGS burden in sub-Saharan Africa. Ultimately, MAP-FGS is expected to provide the essential evidence-base required to support the integration of FGS care into national health policies and routine healthcare systems.

### Programmatic and service delivery

The integration of FGS services within SRH services – including HIV/AIDS, STIs, and cervical cancer – is increasingly recognised as both appropriate and necessary (UNAIDS, 2019; World Health Organization, 2022; Preston et al. 2023; Krentel et al. 2024; Mberu et al. 2025). To support this, a comprehensive prevention, case detection and case management strategy must address each health system building block: (i) leadership and governance; (ii) service delivery; (iii) health system financing; (iv) health workforce; (v) medicines and technologies; and (vi) health information systems. Multiple elements of this approach have been piloted, yet evidence-based global guidance remains a gap to enable scale-up in endemic regions.

An established FGS training curriculum is essential for health workers to provide quality services to those affected. During the COUNTDOWN NTD implementation research project on FGS in Nigeria and Liberia, materials were developed for educating and training health workers to diagnose and manage FGS cases at primary health care settings or to make referrals, as necessary (Nganda et al. 2023; Oluwole et al. 2023; Piotrowski et al. 2023). The FGS Accelerated Scale Together (FAST) package in Ghana, Ethiopia and Madagascar developed core competencies to deliver FGS services, outlining the requirements for all cadres of health workers (Jacobson et al. 2022). These services included community awareness raising, advocacy for prevention through MDA, training for health workers, and diagnosis and treatment services. FAST Package countries have developed training modules to be delivered online or in person and have seen high engagement online from health workers in endemic settings, in addition to FGS training incorporated into national institutional training for healthcare workers (Krentel et al. 2024).

Building on the competencies above, service integration points were identified to inform the development of a minimum service package (MSP) to guide health planners and managers on how to integrate FGS services into routine SRH services, including HIV, cervical cancer and family planning services (Pillay et al. 2024). The MSP focuses on three key service areas: health literacy, screening and diagnosis, and treatment and care. A fourth component was included to promote social inclusion and equity across all three service areas. The MSP has been piloted in Kenya, with data forthcoming on the feasibility, acceptability and cost of implementing the package.

Despite these advances, preventing FGS and reaching women early, prior to the development of irreversible lesions, remains a significant challenge. This is particularly true in the absence of an enabling environment and in the context of declining global support for MDA (Anderson et al. 2023; Lay, 2025). To address this, a pilot project in Côte d’Ivoire integrated presumptive praziquantel treatment and health education into routine SRH services (Preston et al. 2023). This approach reached more than 8,500 at-risk women and led to increased access to preventive chemotherapy for adolescents and women. Health workers reported that they could feasibly incorporate FGS prevention into routine consultations and retained knowledge on key FGS health education messages.

### Community awareness and engagement

Raising awareness and effective engagement regarding FGS within communities supports women and girls in recognising early signs and symptoms, prompting timely health-seeking behaviour and reducing the stigma often associated with reproductive health issues. Several programmes have demonstrated successful approaches to community engagement. The FAST Package, for instance, developed culturally relevant materials such as adolescent storybooks, parent information sheets, and educators’ booklets, tailored specifically for communities in Ghana, Ethiopia and Madagascar (Jacobson et al. 2022; Vlassoff et al. 2022; Krentel et al. 2024). Similarly, both the COUNTDOWN NTDs and Côte d’Ivoire projects produced targeted educational resources designed to increase local knowledge of FGS symptoms and management strategies (Dean et al. 2023; Preston et al. 2023). Drama-based initiatives have also proven valuable, notably in Zambia, Malawi and Tanzania, where community theatre performances effectively communicated messages about FGS prevention and the importance of early diagnosis, significantly enhancing community engagement and treatment uptake (Masong et al. 2021; Ndubani et al. 2025).

### Health management information systems

Lack of data on FGS prevalence and cases, in addition to how affected women interact with the health system - the referral pathways and treatment outcomes - severely limit ministries of health in endemic countries from effectively planning, targeting services and advocating for resources. To date, very little progress has been made on routine reporting of FGS cases, largely due to a lack of training for health workers and the absence of FGS indicators in the HMIS. The Ghana health ministry has inserted genital schistosomiasis indicators into their HMIS with an increase of reported cases, which provide precise data to inform targeted interventions and patient care (Vlassoff et al. 2022; Gyapong et al. 2024; Krentel et al. 2024), and discussions are under way in other countries, for example, Kenya, on similar actions (Karuga et al. 2025). Collecting more FGS data will be essential for future strategy development, and data quality will rely on a coordinated approach with training of health workers, effective diagnostic tools and tailored services.

### Diagnostics and therapeutics advances

Accurate diagnosis of FGS remains challenging, primarily due to limited diagnostic tools and confounding clinical presentations (Søfteland et al. 2021). Visual diagnosis of FGS is considered one of the most clinically effective options. Often colposcopy is used to improve image collection and identification of cervicovaginal lesions, but standard colposcopes are costly, require extensive training and are difficult to deploy in resource-limited settings. Handheld colposcopes, or other portable devices with cameras, offer a more affordable solution (Søfteland et al. 2021). Whilst the absence of standardised clinical protocols hampers implementation, there is progress in developing methodologies for image analysis, including the innovative Digital Gridded Image Technique (DGIT). This has been validated to standardise lesion assessment, although implementation remains resource-intensive (Arenholt et al. 2022; Dragsbæk et al. 2024). Artificial intelligence-assisted image review has promise for simultaneously detecting FGS and cervical abnormalities. However, visual diagnosis relies on clinical presentation of FGS in the lower genital tract, risking that only chronic stages of the disease are identifiable and presentation in the upper genital tract is missed entirely.

Diagnostics for early detection are essential, and there has been progress in recent years in both molecular and syndromic approaches. Recent field trials demonstrate that self-collected genital swabs analysed using polymerase-chain reaction (PCR) improved case detection compared to clinician-collected samples (Sturt et al. 2020b). Furthermore, similar approaches using loop-mediated isothermal amplification (LAMP) and recombinase polymerase amplification (RPA) assays have comparable sensitivity and could be portable options, highlighting potential for decentralised diagnostics in endemic settings (Archer et al. 2022; van Bergen et al. 2024). Various questionnaire-based diagnostic algorithms have been developed, offering a potential solution for use in primary healthcare facilities and resource-limited locations. Although differential diagnosis is more challenging with these approaches, and the testing of these tools remains country-specific (Oluwole et al. 2023; Rogers et al. 2024). Despite these advancements, further progress is required to develop validated diagnostic tools that are adapted and appropriate at the different levels of the health system and to be integrated with existing SRH approaches.

Therapeutic innovations are needed in parallel to developing diagnostics so that there are options for patient management at all stages of case detection. Praziquantel remains the only FGS treatment available, albeit recent studies indicate limited efficacy in resolving established genital lesions. A randomised control trial in Madagascar found no significant improvement in cervical lesions following multiple doses compared to a single standard dose, despite enhanced parasite clearance (Arenholt et al. 2024). These findings reinforce that early and repeated interventions throughout childhood and adolescence are crucial to prevent irreversible genital damage (Preston et al. 2023).

### Policy development

In response to the growing calls for a coordinated, global approach, the WHO is convening multiple departments to form a cross-cutting genital schistosomiasis taskforce. This initiative aims to identify and respond to gaps in policy and programmatic guidance for endemic countries. In 2024, WHO chaired key meetings on FGS at the International AIDS Conference and the World Health Summit (FGS Integration Group, 2024), and in 2025 convened researchers, national coordinators and programme managers in an informal consultation to ‘review evidence, discuss technical updates, identify global priorities and the way forward to address genital schistosomiasis’ (World Health Organization, 2025). A policy brief outlining global priorities and recommendations for FGS integration is expected to be published in 2026, alongside further international consultations to inform strategy and guidance development. Crucially, the WHO African Region’s Expanded Special Project for Elimination of NTDs has secured funding from the German government to improve availability and accessibility of data, coordination and information resources, aiding national NTD programmes to effectively advocate for and integrate FGS with broader health programmes (Expanded Special Project for Elimination of NTDs, 2025).

At the national level, several health ministries in endemic countries are taking the lead in incorporating FGS into policy frameworks. The Ministry of Health, Malawi, for instance, has integrated FGS into its national STI guidelines, which are currently undergoing validation. Similarly, the Ministry of Health and Social Welfare in Liberia included FGS into its NTDs Master Plan 2023–2027 and has developed, in detail, the first cross-sectoral National FGS Strategy. Progress in national policy development has also been aligned with global funding mechanisms. Several countries are incorporating FGS, alongside HIV, into their applications to the Global Fund to leverage resources and strengthen integrated service delivery.

### Creating an enabling environment

As highlighted, these efforts reflect significant progress internationally and within Africa to foster an environment for integrating FGS into broader health frameworks. In 2021–2022, building on ongoing discussions within NTD and SRH rights (SRHR) communities, a coalition of NGOs known as the FGS Integration Group (FIG) was established. FIG aims to facilitate cross-sector collaboration and integrate comprehensive FGS responses into health policies, programmes, and services, ultimately addressing HIV and SRH needs among women and girls. FIG currently comprises 20 member organizations and observers and is actively expanding.

At the national level, several countries have established dedicated coordination groups, including Nigeria’s FGS Society, Ghana’s FGS Committee (established through the FAST Package project), and the National FGS Working Group in Côte d’Ivoire, underscoring growing commitment and collaboration towards tackling FGS comprehensively.

## Critical threats

In recent years, significant shifts in the global health and development landscape have contributed to a more precarious environment for health programme financing and implementation, which could impede efforts to address FGS (Center for Global Development, 2021; Anderson et al. 2023; FGS Integration Group, 2024; Frontline, 2025; Lay, 2025). Reductions in bilateral funding, including significant aid cuts by the United Kingdom and United States governments, have disrupted critical NTD, SRH and child health programmes, threatening to reverse major public health gains. Other countries, such as Australia and the Netherlands, have also reduced overseas development assistance, further shrinking global response to multiple health priorities.

Climate change presents an additional threat by expanding the geographic range of schistosomiasis, increasing transmission risk and burdening already fragile health systems (Klepac et al. 2024). Additionally, emerging infectious diseases, including COVID-19, have disrupted health programmes targeting women and girls, diverted resources, and shifted global health priorities, exacerbating service delivery gaps. At the same time, persistent inequities in healthcare access, compounded by gender biases and economic disparities, continue to marginalise women and girls, limiting their ability to receive timely diagnosis and care (Lucero‐Prisno et al. 2025). Rising healthcare costs, particularly in low- and middle-income countries, pose further barriers to accessing existing limited services for FGS and with broader health challenges, such as mental health disorders associated with chronic disease stigma and disability (Bolton et al. 2023).

These threats, though challenging, offer a unique opportunity to advance collaboration, integrate health approaches, and strengthen domestic resource mobilisation for health services. It has become increasingly clear that the NTD community can no longer address these challenges in isolation.

## Time to act – what will it take?

**Figure 1.**
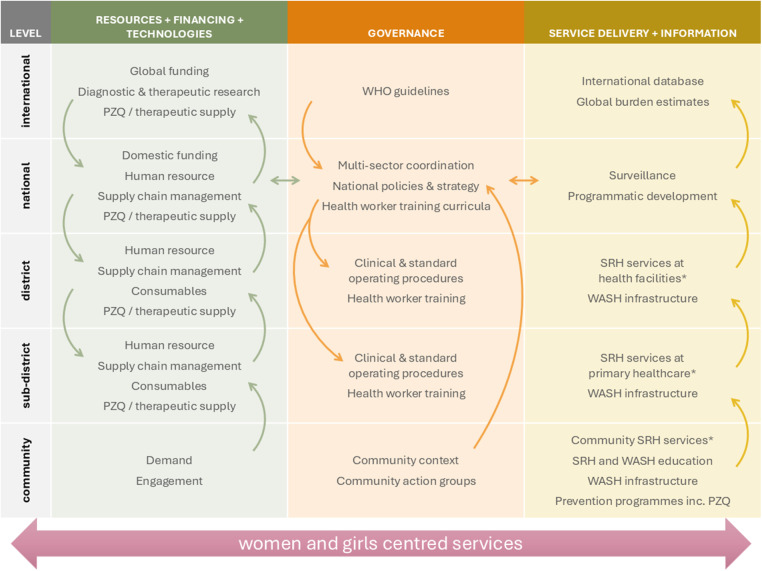
Priority interventions and investments for coordinated comprehensive services to address the neglect of FGS. PZQ, praziquantel; SRH, sexual reproductive health; WASH, water, sanitation and hygiene. *Including appropriate diagnosis and management of FGS dependent on health system level.

Significant international and regional progress has established FGS as a critical SRHR issue, not merely an NTD. Ensuring engagement and demand for comprehensive FGS services from women and girls within communities requires coordinated interventions and investments at multiple levels from the community to the global level (Figure 1). To ensure these efforts are accelerated requires targeted, evidence-based actions with clearly defined strategies.

- ***Position FGS as an SRHR issue***: Explicitly frame FGS within the context of SRHR and gender equity, integrating its management into existing SRH programmes, highlighting FGS as a critical component of comprehensive women’s healthcare. This approach should aim to reduce stigma associated with the disease, reinforce women’s health rights, and ensure equitable healthcare access. Educational campaigns should highlight the links between FGS, gender disparities, and health inequalities, fostering community awareness and empowerment. Communication at all levels should highlight the broader public health benefits associated with addressing FGS, including reducing susceptibility to HIV and cervical cancer.
- ***Integrating FGS prevention into health services and expanding access to praziquantel***: To prioritise early and widespread prevention to prevent women and girls from developing irreversible complications, praziquantel distribution should be embedded across existing health prevention and campaign platforms. This includes continuous availability by integrating praziquantel, including the forthcoming paediatric formulation, into child health visits, immunization programmes, HIV prevention services, and adolescent health services such as HPV vaccination (World Health Organization, 2022). Routine SRH services, including antenatal care, family planning, STIs, cervical cancer, and HIV clinics, should provide FGS screening and on-site praziquantel treatment. Health workers need training and continuous mentoring to recognise FGS symptoms and initiate timely care. Importantly, praziquantel must be consistently stocked in primary healthcare facilities, ensuring preschool-aged children, women and girls can access treatment whenever they present, rather than relying only on annual campaigns. This shift requires reliable supply chains, workforce training, and sustained health system financing.
- ***Strengthen data systems***: Integrate specific FGS indicators into national HMIS, taking lessons from countries like Ghana. Accountability to report against indicators requires community and facility level data capture through, at a minimum, passive surveillance of FGS. Simultaneously, health worker capacity can be enhanced through targeted training, ensuring accurate identification, systematic reporting, and effective utilisation of FGS data. This robust data infrastructure can enable accurate burden estimates to guide resource allocation, programme evaluation, and policy development.
- ***Enhance cross-sectoral collaboration***: Foster and sustain strategic partnerships across SRH, HIV/AIDS, cervical cancer prevention and screening, and water, sanitation, and hygiene (WASH) sectors. Groups like FIG demonstrate the potential for coordinated actions to improve community awareness, reduce transmission, and enhance the integration of preventive measures. Establishing clear communication channels and joint planning mechanisms among stakeholders can optimise these collaborations.
- ***Develop and disseminate normative and technical guidance***: Accelerate the adoption and widespread implementation of WHO guidelines and comprehensive MSP specifically designed for FGS. These guidelines should encompass standardised healthcare worker training, well-defined screening protocols, accessible and practical diagnostic tools, and clear treatment guidelines. Pilot programmes, such as those conducted in Ghana, Ethiopia and Madagascar (FAST), in Kenya (MSP), in Liberia and Nigeria (COUNTDOWN) and in Côte d’Ivoire (SRH integration), can serve as successful models demonstrating feasibility and impact and providing technical insights.
- ***Advance diagnostics, therapeutics and operational research***: Encourage the development and application of innovative multi-pathogen diagnostic tools to improve early detection and integrate FGS screening into routine health services effectively. Explore therapeutic options beyond praziquantel to ensure appropriate management of chronic FGS complications. Utilize evidence generated by ongoing studies, such as the MAP-FGS, to improve diagnostic approaches, inform tailored interventions, identify hotspots, and optimize resource targeting.
- ***Transition to sustainable, integrated health system financing***: Building on the momentum of interest from funders in recent years to support FGS initiatives, efforts should be made to continue backing the remaining research gaps, whilst also transitioning to national health financing models, whereby resource requirements for FGS are embedded with existing SRH and other relevant programmes. There is growing interest among HIV advocates in endemic countries to support the integration of FGS into Global Fund grant applications and domestic resourcing. This should cover comprehensive services for FGS, from prevention to diagnosis and management, and needs to be supported by sustainable and integrated infrastructure, medical equipment and consumables.

The global health community has known for many years that to make this invisible condition visible, we must integrate FGS prevention, diagnosis and management with relevant existing health services, considering all health system building blocks. What has been lacking is coordinated action. In the face of increasing uncertainties in the political and financial landscape for global health, we must galvanise on the need to collaborate as an opportunity to combine the FGS and SRH agenda, and as a commitment to equity and human rights, putting women and girls at the centre.
